# Predictive performance of self-perceived health for depressive symptom development in community-dwelling older adults: a European population-based study

**DOI:** 10.1007/s40520-026-03392-6

**Published:** 2026-04-26

**Authors:** Enrico Ripamonti, Federico Triolo, Amaia Calderón-Larrañaga

**Affiliations:** 1 Department of Economics and Management, University of Brescia, Brescia, Italy; 2 Milan Center for Neuroscience, University of Milan-Bicocca, Milan, Italy; 3 Aging Research Center, Department of Neurobiology, Care Sciences and Society, Karolinska Institutet/Stockholm University, Karolinska Institutet, Stockholm, Sweden; 4 Stockholm Gerontology Research Center, Stockholm, Sweden

**Keywords:** Self-perceived health, Depressive symptoms, SHARE, Aging, Morbidity burden, Mobility, Muscle strength, Cognitive function

## Abstract

**Background:**

Depressive symptoms in later life are common and linked to adverse outcomes, yet early identification remains challenging. Self-perceived health (SPH), a simple subjective measure, may offer prognostic value for depressive symptoms.

**Objective:**

To assess the predictive performance of SPH for depressive symptoms in older adults compared with objective health indicators.

**Methods:**

We analyzed data from 25,985 community-dwelling participants aged 50 + from the Survey of Health, Ageing and Retirement in Europe. Individuals living in nursing homes were excluded. Mean age of participants was 64.4 years (SD = 9.9), 54% were women, and average education was 9.8 years (SD = 4.5). Depressive symptoms were assessed using the 12-item EURO-D scale and defined as a score ≥ 4, in accordance with established cut-offs. Predictive performance of SPH was assessed cross-sectionally and longitudinally over two years by calculating the classification error rate (CER) and the area under the receiver operating characteristic curve (AUC), and compared with number of chronic diseases, cognitive function, grip strength, and walking speed. Analyses were stratified by age and sex.

**Results:**

SPH demonstrated good predictive performance both cross-sectionally and longitudinally. At baseline, SPH had the highest AUC (0.72[0.70,0.73]) and lowest CER (22.4% vs. 24.0% for morbidity burden). At follow-up, performance remained stable (AUC = 0.69[0.67,0.71], CER = 21.8%), comparable to morbidity burden (AUC = 0.64[0.62,0.65], CER = 22.6%), and grip strength (AUC = 0.66[0.64,0.68], CER = 22.3%). Combining SPH with objective indicators modestly improved discrimination.

**Conclusions:**

SPH provides meaningful prognostic information for depressive symptoms in older adults, with performance comparable to objective health measures. Its simplicity supports a potential use in screening and research contexts.

**Supplementary Information:**

The online version contains supplementary material available at 10.1007/s40520-026-03392-6.

## Introduction

 The presence of depressive symptoms in older age is frequent, shaped by a multifaceted interplay of biological, psychological, and social factors. It is not uncommon for depressive symptoms to emerge for the first time during late life [1]. Compared to depressive symptoms in midlife, late-life depressive symptoms may present with a typical clinical picture [2, 3], involve distinct genetic and environmental pathways [4], and are frequently accompanied by comorbid physical and cognitive conditions [5–7]. Risk factors such as chronic illness, functional limitations, cognitive decline, social isolation, and caregiving stressors further contribute to the emergence of these symptoms [7–9]. The cumulative effect of life-course adversity, in line with the accumulation hypothesis [10, 11], may also play a significant role. Depression in old age has been linked to reduced quality of life and even increased non-suicidal mortality [12], highlighting the need for early identification.

In this context, self-perceived health (SPH), namely a person’s own assessment of his/her general health status, has gained attention as a potentially valuable tool for monitoring well-being in aging. SPH typically involves a single-item question reflecting both physical and psychological dimensions of health [13]. SPH reflects an individual’s subjective evaluation of overall health status and integrates information across physical, psychological, and social domains, capturing aspects of health experience that may not be fully represented by objective clinical measures [14, 15]. It is quick to administer, cost-effective, and adaptable to different clinical and research settings. Beyond its practicality, SPH has shown strong associations with a range of health outcomes, including functional status, chronic disease burden, and mental health indicators [16, 17].

The multidimensional nature of SPH supports its role as a holistic indicator of health perception consistent with the biopsychosocial model of health, and suggests its potential utility in mental health research [14]. A growing body of evidence supports the association between poor SPH and depressive symptoms in older adults. Several cross-sectional and longitudinal studies have demonstrated that individuals who perceive their health as poor are at increased risk of maintaining or developing depressive symptoms over time [18–23]. These findings are especially relevant given the challenges in identifying early signs of depression in later life, where people may be less likely to report classical affective symptoms (i.e., low mood) [3] and symptoms may overlap with physical or cognitive complaints. Notably, SPH is routinely and informally assessed in primary care settings, but its appraisal could be useful also in research settings, as a general proxy of (mental) health.

Despite these findings, several key questions remain insufficiently addressed. First, the predictive accuracy of SPH in identifying those at risk for future depressive symptoms, both cross-sectionally and longitudinally, is unclear. Second, there is limited research directly comparing SPH to objective health indicators, such as morbidity burden, in predicting depressive symptoms. Third, it needs to be assessed the differential predictive capacity of SPH across different age and sex groups. Both the prevalence and clinical expression of depressive symptoms depend on age and sex, as well as on health perception and reporting behaviors. For instance, women consistently show higher rates of depressive symptoms, while older individuals may integrate physical decline, multimorbidity, and social losses into their health self-assessment differently than younger individuals. These differences may affect not only risk levels, but also discriminative ability and calibration of predictive models. These knowledge gaps call for further research on the potential of SPH as a prognostic tool for mental health.

This study addressed these gaps by focusing on a large, high-quality, cross-European, population-based cohort of community-dwelling aging individuals. By concentrating on non-institutionalized older adults, we aimed to evaluate the predictive performance of SPH within the general population setting, where early identification strategies are most relevant. Our aim was to assess the predictive accuracy of SPH in identifying the presence and development of depressive symptoms, compared to using objective indicators of health. In doing so, the study evaluated whether incorporating a simple, subjective health measure can sufficiently forecast depressive symptoms in later life, potentially informing age- and sex-specific early screening strategies. This study was designed within a prediction framework, evaluating discrimination and classification performance, rather than testing etiological hypotheses or causal pathways linking SPH to depression. Accordingly, findings should be conceived in terms of prognostic utility and screening potential.

## Methods

### Data and study population

We used data from the Survey of Health, Ageing and Retirement in Europe (SHARE), a longitudinal study of community-dwelling individuals aged 50 years and older. The general aim of the SHARE project is to analyze the process of population aging considering the interactions among medical, psychological, and social factors in the living conditions of the European population aged 50+. It is a prospective cross-national study based on panel databases of individual-level data. From 2004 to 2020, eight longitudinal assessments have been conducted every 2 years. For the present study, we used data from wave 1 (2004) as baseline and wave 2 (2006) as the 2-year follow-up assessment. The SHARE computer-assisted personal interview (CAPI) delves into the health, economic, and social circumstances of the participants. This assessment is conducted repeatedly over time, aiming to capture the evolving characteristics of the aging process. The questionnaires are standardized across different countries to ensure consistency. Eligibility criteria for participation in the SHARE study require individuals to be at least 50 years old, not to have been incarcerated, hospitalized, or out of the country throughout the survey period, and to be proficient in the relevant language(s) of the country or not have relocated to an undisclosed address. Irrespective of their age, current cohabiting partners are also included in the interviews. The longitudinal sample comprises all respondents who participated in any prior survey waves within SHARE.

**Fig. 1:**
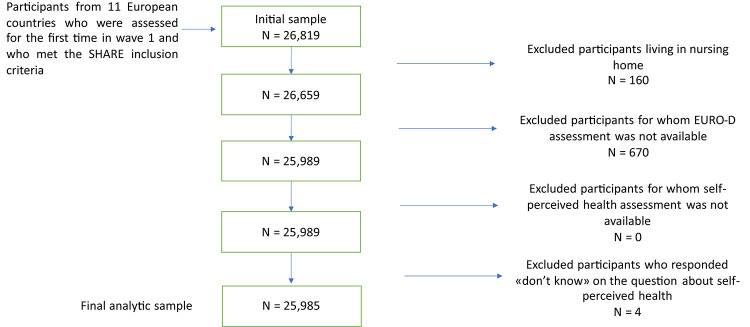
Flowchart illustrating the creation of the analytical sample with inclusion/exclusion criteria

For the purposes of the present investigation, we included data from 11 countries that took part to all SHARE assessments, namely two Nordic European countries (Sweden and Denmark), six Central European countries (Austria, France, Germany, Switzerland, Belgium, The Netherlands), and three Southern European countries (Spain, Italy, and Greece). Length of follow up was 2 years, corresponding to the interval between wave 1 and wave 2. From the included countries, we initially selected 26,819 participants in wave 1 (baseline evaluation). We excluded those who lived in a nursing home (*n* = 160), which led to a sample of 26,659 participants. The rationale for this exclusion criterion is that, compared to community-dwelling individuals, institutionalized older people may have other main drivers for depressive symptoms such as severe diseases or cognitive impairment. We then eliminated participants who did not have the EURO-D assessment available (*n* = 670) and those who responded “don’t know” or had declined to answer the question about SPH (*n* = 4). This led to an analytic sample of 25,985 participants (see Fig. 1), of whom 45.8% were male.

## Measures

*Assessment of depressive symptoms.* At each SHARE interview, the presence of depressive symptoms is evaluated utilizing the EURO-D scale [24]. This tool was designed to standardize the evaluation of depressive symptomatology in later life across European countries. The scale comprises a set of distinct items, encompassing depression, pessimism, suicidality, guilt, sleep patterns, interest, irritability, appetite, fatigue, concentration, enjoyment, and tearfulness. Each of these elements is assigned a score of either 0 (indicating the absence of the symptom) or 1 (indicating the presence of the symptom). The total EURO-D scores can span from 0 to 12. These scores can be treated as a continuous variable or can be categorized based on established criteria. Specifically, the presence of depressive symptoms is identified when a EURO-D score of 4 or higher is recorded [24].

*Assessment of SPH.* SPH is conceptualized as an integrative self-evaluation that reflects how individuals subjectively interpret their physical, psychological, and social well-being within their life context, rather than solely the presence or absence of clinical diagnoses [15]. This global assessment has shown predictive validity for a range of health outcomes, including depressive symptoms, general mortality, and chronic disease trajectories in population studies [15, 25]. In SHARE, SPH is assessed through a single item, where respondents rate their present general health on a five-point Likert scale (see the Supplementary Information). The answer categories are based on the SF-36 questionnaire [26], and comprise the following levels: Excellent; Very good; Good; Fair; Poor. SPH assessment was treated as a continuous score in our analyses. We treated SPH as a continuous variable in our predictive models to preserve the full range of variation in self-rated health perceptions across individuals and to maximize statistical power, consistent with previous work showing linear association of continuous SPH scores with health outcomes [25].

*Objective indicators of health.* Morbidity burden was assessed as the number of chronic diseases, which was operationalized as a continuous variable. In SHARE, information on chronic diseases is obtained through self-report. Participants are asked whether a doctor has ever told them that they have any of a list of 14 conditions, including hypertension, diabetes, heart attack, stroke, cancer, chronic lung disease, arthritis, and others. Cognitive function was measured using the average of two memory tasks: immediate and delayed recall of a 10-word list [27]. In this test, participants first listen to a sequence of 10 words read aloud. They are then asked to recall as many words as possible immediately (first trial) and again after a delay. Each task is scored from 0 to 10, based on the number of correctly recalled words, with higher scores indicating better memory performance. Grip strength was assessed using a handheld dynamometer, with two alternating measurements taken for each hand [28]. The final variable reflects the highest value recorded across all attempts. Only participants with two valid readings per hand are included, and the data is retained only if the two measurements from the same hand differ by no more than 20 kg. Walking speed was evaluated by timing how long it took for participants to walk 2.5 m [29]. This test was conducted twice for each respondent, and the final measure reflects the average of the two trials. Only participants aged 75 years and older were tested for walking speed, and those whose walking times fell outside the 0.54–30 s rage were excluded.

### Statistical methods

The characteristics of the cohort were reported using descriptive metrics such as mean, standard deviation (SD), frequency, and percentage. To evaluate the ability of SPH to predict the presence of depressive symptoms, we used logistic regression. The dataset was randomly split into a training set (80% observations) and a test set (20% observations). A logistic regression model was fit using the training set, with presence of depressive symptoms (binary outcome) as the dependent variable and SPH as the independent variable. Predictive performance was evaluated by calculating the classification error rate (CER) in the test set using a 0.5 probability threshold. Model discrimination was further assessed using the area under the receiver operating characteristic (ROC) curve (AUC) derived from the test set. Non-parametric bootstrap percentile confidence intervals (CI) for AUC were calculated. Monte Carlo cross-validation was applied, i.e., the procedure outlined above was iterated for 1,000 random replicates and averaged [30]. Bootstrap resampling for the computation of AUC CI was performed exclusively on the test sets within each Monte Carlo iteration. This technique was then repeated considering presence of depressive symptoms (dichotomized) at the two-year follow-up. For the longitudinal analyses, only baseline predictors (e.g., SPH at baseline) were used to predict depressive symptoms, ensuring that no follow-up information was included in model training. The same analytic strategy was used to calculate the predictive performance of morbidity burden, cognitive function, muscle strength, and mobility, and of SPH combined with these objective indicators of health. The analyses were stratified by sex (males, females) and age (50–59 yrs., 60–74 yrs., 75 + yrs.), given the expected differences in the prevalence of depressive symptoms across these subgroups. We were only able to compare the predictive performance of walking speed to that of the other indicators within the subsample of those aged 75+. Because CER depends on outcome prevalence and on the chosen classification threshold, CER was only used to compare model performance within groups (e.g., subjective vs. objective health indicators). Between-group comparisons from stratified analyses were interpreted primarily in terms of AUC. Differences between AUCs were assessed qualitatively by comparing point estimates and confidence intervals. Given the exploratory nature of these analyses, no formal statistical tests were performed, and no interaction terms were formally tested. Therefore, stratified comparisons should be deemed as descriptive rather than inferential.

We also conducted a sensitivity analysis considering only participants who at baseline did not present with depressive symptoms, in order to reduce potential bias from baseline symptom status.

The study was reported in accordance with the Strengthening the Reporting of Observational studies in Epidemiology guidelines [31]. All the analytical procedures were carried out utilizing the R software.

## Results

### Descriptive analysis

Participants (Table 1) were on average in their mid-sixties, with slightly more women than men. Overall, the cohort was characterized by moderate educational attainment and relatively low-to-moderate burden of chronic conditions. Approximately one-quarter of participants screened positive for depressive symptoms at baseline. Self-perceived health was generally rated as good or better by the majority of respondents, although a substantial proportion reported fair or poor health. Health-related behaviors showed that most participants were non-smokers or former smokers, and frequent alcohol consumption was reported by a minority. Detailed descriptive statistics for all variables are presented in Table 1 (see also E-Tables 1 and 2).

**Table 1:** Descriptive information for the selected sample at baseline (*n* = 25,985) and stratified by self-perceived health (SPH)

Variable		Self-perceived health (SPH)				
	Whole sample *n* = 25,985	Excellent *n* = 2,649 (10.19%)	Very good *n* = 5,306 (20.42%)	Good *n* = 10,435 (40.16%)	Fair *n* = 5,920 (22.78%)	Poor *n* = 1,675 (6.45%)
*Gender* ^§^						
Male	11,910 (45.83%)	1,391(52.51%)	2,589(48.79%)	4,777(45.78%)	2,462(41.59%)	691(41.25%)
Age^§§^	64.41 (9.94)	60.46(8.39)	61.54(8.89)	64.45(9.72)	67.34(10.13)	69.14(10.75)
Education (yrs) ^§§^	9.83 (4.50)	11.62(3.96)	11.16(4.12)	9.81(4.34)	8.52(4.53)	7.52(4.88)
Number chronic diseases^§§^	1.52 (1.42)	0.57(0.83)	0.85(0.97)	1.41(1.20)	2.31(1.44)	3.11(1.81)
BMI^§§^	25.96 (5.46)	25.10(4.23)	25.45(4.39)	26.05(5.26)	26.46(6.34)	26.60(7.38)
*Presence of depressive symptoms*^§^ *(EUROD scale)*						
Yes	6,420 (24.71%)	217(8.19%)	581(10.95%)	2,101(20.13%)	2,384(40.27%)	1,137(67.88%)
No	19,565 (75.29%)	2,432(91.81%)	4,725(89.05%)	8,334(79.87%)	3,536(59.73%)	538(32.12%)
*Current smoking* ^§^						
Yes, currently smoke	5,012 (19.29%)	550(20.76%)	1,138(21.45%)	1,999(19.16%)	1,019(17.21%)	306(18.27%)
Never smoked daily for at least one year	13,561 (52.19%)	1,234(46.58%)	2,642(49.79%)	5,474(52.46%)	3,300(55.74%)	911(54.39%)
No, I have stopped	7,409 (28.51%)	865(32.65%)	1,524(28.72%)	2,961(28.38%)	1,601(27.04%)	458(27.34%)
Don’t know	3 (0.01%)	0(0.00%)	2(0.04%)	1(0.01%)	0(0.00%)	0(0.00%)
*Drinking* ^§^						
Drinking 2 glasses 5–6 days a week or every day	3,601 (13.86%)	400(15.10%)	749(14.12%)	1,519(14.56%)	732(12.36%)	201(12.00%)
Not drinking more than 2 glasses daily or 5–6 a week	22,359 (86.05%)	2,248(84.86%)	4,552(85.79%)	8,903(85.32%)	5,184(87.57%)	1,472(87.88%)
Don’t know	23 (0.09%)	1(0.04%)	5(0.09%)	11(0.11%)	4(0.07%)	2(0.12%)
Refusal	2 (0.01%)	0(0.00%)	0(0.00%)	2(0.02%)	0(0.00%)	0(0.00%)

^§^ N (percentage); ^§§^ Mean (SD)

### Predictive performance

Table 2 shows the predictive performance of SPH and other objective indicators of health for depressive symptoms at baseline and over the 2-year follow-up. At baseline, self-perceived health (SPH) was the strongest individual predictor: SPH alone yielded the lowest CER (22.4%) and the highest AUC (0.716[0.701,0.730]) among all individual predictors, indicating relatively good discrimination and predictive ability for presence of depressive symptoms. Grip strength and number of chronic diseases performed moderately well (AUCs of 0.668[0.651,0.683] and 0.659[0.643,0.674], respectively). Adding SPH to other predictors consistently improved classification. In particular, combining SPH and grip strength led to AUC = 0.739[0.725,0.754] and a CER of 21.5%.

**Table 2:** Predictive accuracy of self-perceived health (SPH) for presence of depressive symptoms, as compared to objective indicators of health. Analyses for the whole population and stratified by sex. All predictors have been evaluated at SHARE baseline. Depressive symptoms have been evaluated at SHARE baseline, and 2 years from baseline

	Whole sample (n = 25,985)		Male (n = 11,910)		Female (n = 14,075)	
	CER	AUC	CER	AUC	CER	AUC
*Cross-sectional prediction*						
SPH	22.4%	0.716 [0.701, 0.730]	15.3%	0.730[0.705, 0.754]	28.4%	0.707[0.691, 0.723]
N chronic diseases	24.0%	0.659 [0.643, 0.674]	16.1%	0.655[0.630, 0.679]	30.2%	0.655[0.636, 0.673]
Cognitive function	24.7%	0.614 [0.598, 0.629]	16.3%	0.623[0.593, 0.650]	31.3%	0.624[0.605, 0.644]
Grip strength	23.6%	0.668 [0.651, 0.683]	15.5%	0.603[0.574, 0.633]	29.7%	0.624[0.602, 0.645]
SPH +						
N chronic diseases	22.2%	0.731[0.716, 0.746]	15.6%	0.742[0.718, 0.767]	27.4%	0.722[0.706, 0.759]
Cognitive function	22.3%	0.728[0.713, 0.742]	15.3%	0.742[0.717, 0.768]	27.6%	0.723[0.704, 0.741]
Grip strength	21.5%	0.739[0.725, 0.754]	15.1%	0.727[0.701, 0.753]	27.2%	0.714[0.696, 0.734]
*Longitudinal prediction*						
SPH	21.8%	0.688[0.670, 0.706]	15.3%	0.699[0.669, 0.731]	27.3%	0.677[0.656, 0.699]
N chronic diseases	22.6%	0.636[0.616, 0.654]	15.3%	0.621[0.588, 0.654]	28.2%	0.635[0.611, 0.658]
Cognitive function	22.7%	0.597[0.577, 0.618]	15.3%	0.620[0.584, 0.656]	29.0%	0.601[0.577, 0.626]
Grip strength	22.3%	0.662[0.643, 0.682]	14.9%	0.617[0.580, 0.632]	28.2%	0.616[0.589, 0.641]
SPH +						
N chronic diseases	21.9%	0.701[0.682, 0.720]	15.2%	0.706[0.673, 0.737]	26.9%	0.692[0.670, 0.714]
Cognitive function	21.7%	0.700[0.680, 0.718]	15.0%	0.717[0.684, 0.751]	26.9%	0.690[0.666, 0.713]
Grip strength	21.0%	0.720[0.702, 0.737]	14.7%	0.712[0.678, 0.746]	26.3%	0.689[0.665, 0.712]

SPH = self-perceived health

CER = classification error rate

AUC = area under the curve

[] indicates 95% confidence intervals

**Table 3:** Predictive accuracy of self-perceived health (SPH) for presence of depressive symptoms, as compared to objective indicators of health. Analysis stratified by age group. All predictors have been evaluated at SHARE baseline. Depressive symptoms have been evaluated at SHARE baseline, and 2 years from baseline

	50–59 yrs (n = 9,807)		60–74 yrs (n = 11,544)		75 + yrs (n = 4,634)	
	CER	AUC	CER	AUC	CER	AUC
*Cross-sectional prediction* ^§^						
SPH	21.2%	0.698[0.674, 0.722]	21.1%	0.717[0.695, 0.737]	28.0%	0.717[0.690, 0.744]
N chronic diseases	22.2%	0.649[0.624, 0.673]	22.2%	0.662[0.638, 0.686]	31.7%	0.634[0.606, 0.666]
Cognitive function	22.5%	0.565[0.537, 0.593]	22.8%	0.609[0.584, 0.632]	31.2%	0.647[0.615, 0.677]
Grip strength	22.3%	0.654[0.627, 0.680]	22.0%	0.665[0.640, 0.691]	29.2%	0.679[0.644, 0.711]
Walking speed	NA	NA	NA	NA	30.4%	0.653[0.609, 0.697]
SPH +						
N chronic diseases	21.1%	0.713[0.688, 0.738]	21.0%	0.736[0.713, 0.758]	27.6%	0.732[0.703, 0.760]
Cognitive function	21.2%	0.703[0.677, 0.729]	21.1%	0.732[0.710, 0.753]	26.2%	0.746[0.717, 0.774]
Grip strength	20.6%	0.728[0.702, 0.753]	20.3%	0.747[0.725, 0.767]	26.2%	0.750[0.720, 0.779]
Walking speed	NA	NA	NA	NA	27.4%	0.713[0.670, 0.758]
*Longitudinal prediction* ^§§^						
SPH	20.0%	0.676[0.643, 0.705]	20.4%	0.682[0.654, 0.708]	30.6%	0.684[0.647, 0.725]
N chronic diseases	20.0%	0.617[0.584, 0.649]	20.8%	0.635[0.605, 0.663]	33.6%	0.613[0.572, 0.656]
Cognitive function	20.2%	0.543[0.502, 0.580]	20.8%	0.594[0.562, 0.626]	33.4%	0.618[0.572, 0.662]
Grip strength	20.0%	0.637[0.603, 0.671]	20.6%	0.658[0.629, 0.688]	31.8%	0.663[0.616, 0.705]
Walking speed	NA	NA	NA	NA	32.7%	0.601[0.538, 0.663]
SPH +						
N chronic diseases	19.8%	0.685[0.650, 0.717]	20.2%	0.695[0.667, 0.722]	30.5%	0.698[0.658, 0.741]
Cognitive function	20.0%	0.678[0.644, 0.711]	20.2%	0.695[0.664, 0.723]	29.7%	0.707[0.668, 0.745]
Grip strength	19.3%	0.702[0.668, 0.733]	19.7%	0.719[0.691, 0.746]	28.9%	0.721[0.678, 0.759]
Walking speed	NA	NA	NA	NA	30.0%	0.678[0.620, 0.735]

SPH = self-perceived health

[] indicates 95% confidence intervals

When measuring EURO-D scores at the 2-year follow-up, SPH remained the strongest individual predictor: it again showed the lowest CER (21.8%) and the highest AUC (0.688[0.670,0.706]) among individual predictors. Grip strength (AUC = 0.662[0.643,0.682]) and number of chronic diseases (AUC = 0.636[0.616,0.654]) showed moderate predictive value. Cognitive function had the weakest longitudinal predictive performance (AUC = 0.597[0.577,0.618]), indicating limited value for forecasting depressive symptoms over time when used alone. Combining SPH with objective indicators improved prediction. Again, combining SPH and grip strength had the best overall longitudinal performance, with lowest CER (21.0%) and highest AUC (0.720[0.702,0.737]).

### Analysis stratified by sex

When stratified by sex, the predictive performance of the majority of the models appeared numerically higher in men than women, both cross-sectionally and longitudinally. These differences are based on descriptive comparisons of AUC estimates and confidence intervals, as no formal statistical tests of interaction were conducted. In cross-sectional analyses, SPH was the strongest individual predictor in both sexes, but performance was notably better in men (AUC = 0.730[0.705,0.754]) compared to women (AUC = 0.707[0.691,0.723]). For other predictors, we found substantially comparable AUC for males and females. Combining SPH with additional predictors led to further improvements, especially in men. The best-performing cross-sectional model in men was SPH+cognitive function (AUC = 0.742[0.717,0.768]); in women, the same model attained AUC = 0.723[0.704,0.741]. A similar pattern emerged in longitudinal analyses. In men, SPH alone remained a good predictor of depressive symptoms at two years (AUC = 0.699[0.669,0.731]), and the best-performing combination was SPH+cognitive function (AUC = 0.717[0.684,0.751]). Predictive accuracy in women remained lower (e.g., SPH: AUC = 0.677[0.656,0.699]), and combining predictors yielded only modest gains.

### Analysis stratified by age group

In age-stratified analyses, across all age groups, SPH remained the strongest individual predictor of depressive symptoms in both cross-sectional and longitudinal models. The AUC for SPH was relatively stable across age groups (e.g., 0.698[0.674,0.722] in 50–59 years, 0.717[0.695,0.737] in 60–74 years, and 0.717[0.690,0.744] in 75 + years). Performance of the number of chronic diseases had similar AUCs in the 50–59 and 60–74 age groups (0.649[0.624,0.673] and 0.662[0.638,0.686], respectively) but was less predictive in those aged 75+ (AUC = 0.634[0.606,0.636]). Cognitive function showed relatively poor performance in all age groups but was notably weakest in the youngest group (AUC = 0.565[0.537,0.593]), suggesting a limited cross-sectional predictive value for depressive symptoms in middle-aged adults. When SPH was combined with other predictors, performance improved across all age groups. The best cross-sectional model in each age group was SPH+grip strength, with AUCs ranging from 0.728[0.702,0.753] (50–59 years) to 0.750[0.720,0.779] (75 + years). In the oldest age group, walking speed also contributed modestly when combined with SPH (AUC = 0.713[0.670,0.758]). Longitudinal prediction followed a similar pattern, with higher predictive accuracy in the oldest age group (e.g., SPH: AUC = 0.684[0.647,0.725]) compared to younger groups (SPH AUCs: from 0.676 to 0.682). The best longitudinal model in each age group again involved SPH+grip strength, achieving the highest AUC (from 0.702 to 0.721) (see Table 3).

### Sensitivity analysis

To test the robustness of our findings, we conducted a sensitivity analysis restricted to participants who, at baseline, had EURO-D scores below threshold (*n* = 19,565). At the two-year follow-up, SPH remained a meaningful predictor of depressive symptoms, with an AUC of 0.634[0.607,0.661] and a CER of 12.6% (Table 4). Among the individual objective predictors, grip strength showed the highest discriminative performance (AUC = 0.620[0.590,0.649]; CER:12.4%), while cognitive function had the lowest predictive performance (AUC = 0.566[0.534,0.598]). When combining SPH with objective indicators, overall predictive performance improved modestly. The best-performing model was the combination of SPH and grip strength, yielding an AUC of 0.663[0.634,0.691] and a CER of 12.4%.

**Table 4:** Sensitivity analysis. Predictive accuracy of self-perceived health (SPH) for presence of depressive symptoms, as compared to objective indicators of health in those without depressive symptoms at baseline (*n* = 19,565)

	CER	AUC
Longitudinal prediction^§§^		
SPH	12.6%	0.634[0.607, 0.661]
N chronic diseases	12.6%	0.601[0.574, 0.629]
Cognitive function	12.6%	0.566[0.534, 0.598]
Grip strength	12.4%	0.620[0.590, 0.649]
SPH +		
N chronic diseases	12.6%	0.647[0.620, 0.675]
Cognitive function	12.6%	0.643[0.615, 0.671]
Grip strength	12.4%	0.663[0.634, 0.691]

SPH = self-perceived health[] indicates 95% confidence intervals

## Discussion

In this large, European, population-based cohort of older adults, we found that SPH is a robust predictor of depressive symptoms, performing similarly or outperforming other objective health indicators. These findings should be interpreted within a predictive framework, as our analyses evaluated discriminative performance rather than causal effects. Moreover, while CER provides an intuitive measure of overall predictive accuracy, its magnitude depends on the prevalence of depressive symptoms in the sample and on the chosen probability threshold (0.5 in our analyses). Therefore, CER should be understood in relation to the specific study context rather than as an absolute indicator of model performance.

SPH demonstrated relatively good predictive performance both cross-sectionally and over a two-year follow-up period, with consistent but different results across sex and age strata. While other indicators, such as morbidity burden and muscle strength, also showed moderate predictive value, they did not match the overall performance of SPH. The objective measures, particularly grip strength, were similar to SPH in the subsample without depressive symptoms at baseline. Cognitive function and mobility were comparatively weaker predictors, particularly in longitudinal models. Importantly, combining SPH with objective indicators consistently improved predictive performance, especially when paired with grip strength. This picture supports the clinical and research utility of SPH as a simple, accessible tool for identifying individuals at risk of depressive symptoms in later life.

Our findings, together with those from previous studies [32, 33], suggest that SPH may capture multidimensional aspects of health that extend beyond the scope of physical or cognitive measures alone. As a subjective assessment, SPH likely reflects an individual’s integration of physical functioning, emotional distress, and social limitations [34], many of which are relevant to the onset or maintenance of depressive symptoms. Socioeconomic status has been also described as a possible moderator of the role of SPH [35].

Stratified analyses revealed that, in absolute and descriptive terms, the predictive accuracy of SPH at baseline was higher for men (AUC:0.730) than women (AUC:0.707). The same was true at follow-up (AUC:0.699 vs. 0.677). This discrepancy may reflect differences in symptom reporting, biological vulnerability, or psychosocial factors such as coping strategies and social roles [36, 37]. Interestingly, the sex gap in model performance persisted even when combining SPH with objective indicators, underscoring the need for further research into sex-specific determinants and manifestations of late-life depressive symptoms.

SPH proved to be a robust predictor of depressive symptoms across all age groups, with only modest variations in predictive accuracy. This pattern was found in both cross-sectional and longitudinal analyses. Interestingly, while the discriminative ability of morbidity burden tended to decline with age, SPH remained stable or even slightly stronger among the oldest participants. This pattern suggests that older adults may integrate a broader range of information, including emotional, social, and functional aspects, when assessing their health, thus making SPH an increasingly comprehensive indicator of well-being in later life. The predictive performance of SPH, also in combination with other predictors, was lower in the group aged 50–59 years, indicating that in the transition phase subjective judgements about health can contain relatively heterogeneous information, which might be challenging to reliably relate to mental health outcomes. Particularly in this phase it is important to integrate information on SPH with that of objective indicators. Our results reinforce the potential of SPH as an accessible and valid measure to monitor depressive risk throughout the aging process, but also emphasize the need for age-tailored interpretations, given that the meaning and correlates of SPH may shift with different ages.

The finding that SPH was the single strongest predictor of concurrent and 2-year depressive symptoms is consistent with longitudinal evidence showing that poorer self-rated health prospectively predicts increases in depressive symptomatology for older adults, and that the relationship may vary by age [38]. Persistent female excess in depressive symptoms and presence of different predictors according to sex may explain why predictive models in our sample performed better in men than women [39]. With respect to objective measures, our finding that lower grip strength adds predictive value to SPH, but modest predictive effect when considered alone, could align with previous results [40]. Similarly, prior work indicates that slower gait predicts incident depressive symptoms [41], which is in line with our observation that walking speed contributed modestly but discernibly within the 75+ subsample. The relatively weak predictive performance of the episodic memory measure in our longitudinal models is consistent with prospective studies reporting only small or age-dependent effects of memory on later depressive symptoms together with evidence of bidirectional, though modest, links of memory and depression [42]. This may suggest that single-domain cognitive tests have limited standalone prognostic value for future mood changes.

SPH is widely collected in epidemiological research as well as in clinical practice, yet its value as a screening tool remains underutilized. Our study shows that SPH is not only simple and feasible to gather, but also informative for predicting depressive symptoms. It may be particularly useful in research settings where formal diagnostic tools are unavailable, or as an early signal prompting further mental health evaluation in clinical settings. Moreover, our findings suggest that integrating SPH into predictive models with selected objective indicators (e.g., grip strength) may enhance early risk identification strategies [43]. These models could be readily incorporated into geriatric screening protocols, public health surveys, or digital health tools aimed at monitoring mental well-being in older populations.

Key strengths of this study include the use of a large, diverse sample from the SHARE study, standardized assessments of depressive symptoms across countries, and rigorous validation methods including cross-validation and bootstrap confidence intervals. Additionally, the inclusion of both cross-sectional and longitudinal predictions strengthens the temporal validity of our findings. However, some limitations should be acknowledged. First, the use of a single-item SPH measure, while pragmatic, may not capture nuanced aspects of subjective health perceptions. Although participants report physician-diagnosed conditions, this measure remains based on self-report and may therefore be subject to reporting inaccuracies or recall bias. More broadly, several variables in this study were based on self-report, including morbidity burden, smoking, and alcohol consumption. As such, they might be subject to recall bias and social desirability bias. Participants may underreport stigmatized conditions or unhealthy behaviors, or may differently recall past diagnoses depending on cognitive status or educational level. These reporting tendencies could have introduced non-differential or differential measurement error, potentially affecting the observed predictive performance of both subjective and objective indicators. Second, depressive symptoms were assessed via the EURO-D scale, which, while validated, is a screening tool rather than a clinical diagnosis. Third, walking speed was only assessed in participants aged 75 and older, limiting comparability across age groups. As a result, the contribution of mobility to depressive symptom prediction could only be examined in the oldest subgroup, and its relative performance compared to other predictors across the full age spectrum should be interpreted with caution. Fourth, our findings are limited to community-dwelling older adults and may not generalize to institutionalized populations, such as nursing home residents, who may have different determinants and trajectories of depressive symptoms.

In conclusion, this study provides robust evidence that SPH may be a valuable predictor of depressive symptoms in older adults. Its predictive performance is comparable to, or even exceeds, that of objective measures of physical and cognitive health. Given its simplicity, accessibility, and predictive power, SPH should be considered a core component of mental health risk assessment in aging populations. Future studies should examine the integration of SPH into clinical practice and its broader applicability for predicting mental health outcomes in aging populations.

## Supplementary Information

Below is the link to the electronic supplementary material.

Supplementary Material 1

## Data Availability

No datasets were generated or analysed during the current study.
