# Supplementary material for: Predictive performance of self-perceived health for depressive symptom development in community-dwelling older adults: a European population-based study
Source: Aging Clin Exp Res. 2026 Apr 26;38(1):115. doi: 10.1007/s40520-026-03392-6 (PMC13121397; doi:10.1007/s40520-026-03392-6)
Supplement: Supplementary file 1 — Supplementary Material 1 [file 40520_2026_3392_MOESM1_ESM.docx]

**Supplementary information**

Predictive performance of self-perceived health for depressive symptom development in community-dwelling older adults: A European population-based study

**Self-perceived health – US version**

In this study, we used the variable sphus (self-perceived health, US version). The variables sphus and spheu (self-perceived health, European version) capture self-perceived health using a single-item measure. Participants are asked to evaluate their current overall health status using a five-point Likert-type scale. The European and US versions differ in wording and range of response categories. In the European version (spheu), response options range from “very good” to “very poor”. In contrast, the US version (sphus), derived from the SF-36 health survey (Ware and Gandek, 1998), uses response categories ranging from “excellent” to “poor”. In Wave 1 of SHARE, both the European and the US versions of the self-perceived health question were administered. Depending on the interview structure, respondents answered the item either at the beginning or at the end of the physical health (PH) module. From Wave 2 onward, only the US version of the self-perceived health item has been included in SHARE. For a detailed comparison between the European and US versions, see Jürges et al. (2008).

Response options are coded as: 1: Excellent; 2: Very good; 3: Good; 4: Fair; 5: Poor

**References**

Jürges H, Avendano M, Mackenbach JP. Are different measures of self-rated health comparable? An assessment in five European countries. Eur J Epidemiol 23: 77-781, 2008.

Mehrbrodt T, Gruber S, Wagner M. SHARE Scale and multi-item indicators. SHARE Consortium, 2019.

Ware JE, Gandek B. Overview of the SF-36 health survey and the international quality of life assessment (IQOLA) project. J Clin Epi 51(11): 903-912, 1998.

**E- Tables**

**E-Table 1.** Descriptive information for the selected sample at baseline (N = 25,985) and stratified by sex.

| **Variable** | **Whole sample**  **N = 25,985** | **Only male**  **N = 11,910** | **Only female**  **N = 14,075** |
| --- | --- | --- | --- |
| *Gender*^§^ |  |  |  |
| Male | 11,910 (45.83%) |  |  |
| Age^§§^ | 64.41 (9.94) | 64.17 (9.61) | 64.61 (10.21) |
| Education (yrs) ^§§^ | 9.83 (4.50) | 10.45 (4.52) | 9.31 (4.42) |
| Number chronic diseases^§§^ | 1.52 (1.42) | 1.40 (1.34) | 1.63 (1.48) |
| BMI^§§^ | 25.96 (5.46) | 26.50 (4.40) | 25.51 (6.17) |
| *Presence of depressive symptoms*^§^ *(EUROD scale)* |  |  |  |
| Yes | 6,420 (24.71%) | 1,945 (16.33%) | 4,475 (31.79%) |
| No | 19,565 (75.29%) | 9,965 (83.67%) | 9,600 (68.21%) |
| *Self-perceived health*^§^ |  |  |  |
| Excellent | 2,649 (10.19%) | 1,391 (11.68%) | 1,258 (8.94%) |
| Very good | 5,306 (20.42%) | 2589 (21.74%) | 2,717 (19.30%) |
| Good | 10,435 (40.16%) | 4,777 (40.11%) | 5,658 (40.20%) |
| Fair | 5,920 (22.78%) | 2,462 (20.67%) | 3,458 (24.57%) |
| Poor | 1,675 (6.45%) | 691 (5.80%) | 984 (6.99%) |
| *Current smoking*^§^ |  |  |  |
| Yes, currently smoke | 5,012 (19.29%) | 2,820 (23.68%) | 2,192 (15.57%) |
| Never smoked daily for at least one year | 13,561 (52.19%) | 4,104 (34.46%) | 9,457 (67.19%) |
| No, I have stopped | 7,409 (28.51%) | 4,984 (41.85%) | 2,425 (17.23%) |
| Don’t know | 3 (0.01%) | 2 (0.02%) | 1 (0.01%) |
| *Drinking*^§^ |  |  |  |
| Drinking 2 glasses 5-6 days a week or every day | 3,601 (13.86%) | 2,676 (22.47%) | 925 (6.57%) |
| Not drinking more than 2 glasses daily or 5-6 a week | 22,359 (86.05%) | 9,218 (77.40%) | 13,141 (93.36%) |
| Don’t know | 23 (0.09%) | 15 (0.13%) | 8 (0.06%) |
| Refusal | 2 (0.01%) | 1 (0.01%) | 1 (0.01%) |

^§^ N (percentage); ^§§^ Mean (SD)

**E-Table 2.** Descriptive information for the selected sample at baseline (N = 25,985) and stratified by presence of depressive symptoms.

|  |  | **Presence of depressive symptoms** | |
| --- | --- | --- | --- |
| **Variable** | **Whole sample**  **N = 25,985** | **Yes**  **N = 6,420** | **No**  **N = 19,565** |
| *Gender*^§^ |  |  |  |
| Male | 11,910 (45.83%) | 1,945(30.30%) | 9,965(50.93%) |
| Age^§§^ | 64.41 (9.94) | 66.05(10.89) | 63.87(9.54) |
| Education (yrs) ^§§^ | 9.83 (4.50) | 8.42(4.65) | 10.29(4.35) |
| Number chronic diseases^§§^ | 1.52 (1.42) | 2.17(1.65) | 1.31(1.27) |
| BMI^§§^ | 25.96 (5.46) | 25.83(6.75) | 26.01(4.96) |
| *Self-perceived health*^§^ |  |  |  |
| Excellent | 2,649 (10.19%) | 217(3.38%) | 2,432(12.43%) |
| Very good | 5,306 (20.42%) | 581(9.05%) | 4,725(24.15%) |
| Good | 10,435 (40.16%) | 2,101(32.73%) | 8,334(42.60%) |
| Fair | 5,920 (22.78%) | 2,384(37.13%) | 3,563(18.07%) |
| Poor | 1,675 (6.45%) | 1,137(17.71%) | 538(2.75%) |
| *Current smoking*^§^ |  |  |  |
| Yes, currently smoke | 5,012 (19.29%) | 1,193(24.03%) | 3,819(19.52%) |
| Never smoked daily for at least one year | 13,561 (52.19%) | 3,584(57.38%) | 9,877(50.48%) |
| No, I have stopped | 7,409 (28.51%) | 1,543(24.03%) | 5,866(29.98%) |
| Don’t know | 3 (0.01%) | 0(0.00%) | 3(0.02%) |
| *Drinking*^§^ |  |  |  |
| Drinking 2 glasses 5-6 days a week or every day | 3,601 (13.86%) | 749(11.67%) | 2,852(14.58%) |
| Not drinking more than 2 glasses daily or 5-6 a week | 22,359 (86.05%) | 5,661(88.18%) | 16,698(85.35%) |
| Don’t know | 23 (0.09%) | 8(0.12%) | 15(0.08%) |
| Refusal | 2 (0.01%) | 2(0.03%) | 0(0.00%) |

^§^ N (percentage); ^§§^ Mean (SD)
